# Influence of Altitude on the Hearing and Motion-Sensing Appararatus of the Ear

**Published:** 1919-01

**Authors:** 


**Influence of Altitude on the Hearing and Motion-Sensing Appararatus of the Ear.** Lieut.-Colonel E. R. LEWIS, M. C., N. A. *The Journal of the American Medical Association,* October 26, 1918.

The author records a series of experiments made with a view to determining the effects of altitudes on the ear functions. In these experiments both the Henderson rebreathing apparatus and the decompression tank types of artificial altitude have been utilized. It has been determined that the hearing function shows no deterioration attributable to impairment of the perceptive element prior to attaining a degree of altitude that causes acute general functional impairment of all the higher cerebral sensory and psychic centers.

During ascent from a denser to a rarer atmosphere, the sound-conducting apparatus may show transitory interference with function, attributable to expansion of air within the eustachian tube, tympanic cavity or air spaces of the mastoid resulting in inequality of intratympanic and extratympanic air pressures. Such disturbances are much more marked during rapid descent, when the discrepancy between intratympanic and extratympanic air pressure becomes considerable. In some cases the extratympanic pressure caused perforation of the drumhead. Practically all cases showed congestion of the drumhead following rapid descent, and occasionally the irritation resulting from changes of altitude have been observed to cause acute otitis media.

Observations on the effects of altitude on the motion-sensing function of the ear have revealed nothing in the nature of obtunded ability to perceive motion or alterations in eye reactions to motion stimulus.

